# Blood Mechanical Intelligence: From Force Sensing to Precision Mechanomedicine

**DOI:** 10.34133/research.1157

**Published:** 2026-02-25

**Authors:** Shichun Wang, Ting Pan, Qi Liu, Zedong Li, Ting Wen, Bo Cheng, Feng Xu, Chunyan Yao

**Affiliations:** ^1^ Department of Blood Transfusion, The First Affiliated Hospital (Southwest Hospital) of Army Medical University, Chongqing 400038, P.R. China.; ^2^ PLA Chongqing Blood Center, Chongqing 400038, P.R. China.; ^3^ MOE Key Laboratory of Biomedical Information Engineering, Bioinspired Engineering and Biomechanics Center, School of Life Science and Technology, Xi’an Jiaotong University, Xi’an 710049, P.R. China.; ^4^ Bioinspired Engineering and Biomechanics Center (BEBC), School of Life Science and Technology, Xi’an Jiaotong University, Xi’an 710049, P.R. China.; ^5^ Hainan General Hospital, Hainan Affiliated Hospital of Hainan Medical University, Haikou 570311, P.R. China.

## Abstract

Blood is a force-rich system. Beyond biochemistry, blood cells continually sense shear, stretch, and substrate rigidity, encode these inputs into biochemical and structural state changes, and adjust future behavior. We term this mechanical intelligence: an operational framework encompassing mechanosense, learning and memory, decision-making, and adaptive evolution. We synthesize evidence across red blood cells (RBCs), leukocytes, and platelets showing how mechanosensitive receptors (Piezo-type mechanosensitive ion channel component 1, glycoprotein Ib-IX-V complex, and integrins), cytoskeletal feedback, and nuclear effectors (Yes-associated protein/transcriptional coactivator with PDZ-binding motif and nuclear factor κB) couple mechanical cues to cellular states and functions (oxygen delivery, immune surveillance, and hemostasis). Repeated mechanical exposures produce mechanical memory (e.g., RBC fatigue and shape memory, shear-primed platelet hyperreactivity, and stiffness-modulated leukocyte transmigration) that improves performance in physiological contexts but contributes to pathology when dysregulated (vaso-occlusion, thrombosis, and inflammaging). We outline mechanodiagnostics (single-cell deformability cytometry, atomic force microscopy, and microfluidic thrombus profiling) and mechanotherapy (ion channel modulators, engineering cells, and mechanocompatible devices) and propose reporting standards, readiness levels, and validation pathways.

## Introduction

Recent scientific breakthroughs reveal that cells can exhibit human intelligence, similar to behaviors through dynamic interactions with their microenvironment. These behaviors, including multimodal perception, problem-solving, mechanical memory, and evolutionary adaptation, are collectively termed “cellular intelligence” [1,2]. Subsequently, Cheng et al. [3,4] defined “cellular mechanical intelligence” as follows: Cells or cell clusters sense mechanical microenvironmental signals (shear stress, pressure, adhesive force, etc.), coordinated action of the mechanosensitive molecules, cytoskeleton, and signaling pathways, and achieve autonomous adaptive processes involving signal sensing, memory storage, decision-making, and functional optimization. Its core principle is “mechanically driven active intelligent behavior”. Blood consists of plasma and blood cells (red blood cells [RBCs], leukocytes, and platelets), playing a crucial role in transporting oxygen and nutrients, removing waste products and carbon dioxide, and maintaining homeostasis within the body. The mechanical properties of blood involve viscosity, shear stress, and the stiffness of the microenvironmental matrix. Blood cells continuously respond these mechanical factors within the hemodynamic environment, translating mechanical signals into intracellular biochemical signals that regulate cellular morphology and function [5]. On the basis of these findings, we propose that RBCs, leukocytes, and platelets collectively perform dynamic mechanosensory functions, fundamentally redefining the blood as an intelligent mechanical system.

This paradigm shift is rooted in insights from across hematology, immunology, physics, and bioengineering. A key breakthrough was the discovery of mechanosensitive ion channels (exemplified by Piezo-type mechanosensitive ion channel component 1 [PIEZO1]) that convert membrane tension into biochemical signals [6]. Such channels, first identified a decade ago in nonblood cells [4,7], were soon found in blood cell lineages as well [8]. For example, in RBCs, mechanical activation of PIEZO1 triggers Ca^2+^ influx that regulates cell volume and deformability, while mutations in the PIEZO1 gene cause a hereditary anemia characterized by dehydrated and rigid RBCs [9], directly implicating mechanotransduction in blood physiology. Mechanical intelligence in blood also extends to the immune system. Leukocytes monitor and respond to biophysical cues such as fluid shear stress in the bloodstream and matrix stiffness in tissues, as they circulate or migrate through the tissues [10]. They also require mechanical forces at the immunological synapse to fully activate, where the T cell receptor (TCR) behaves as a mechanosensor that enhances antigen discrimination under force [11]. This emerging mechanoimmunology underscores that immune cells integrate both chemical and physical information to make decisions [12]. Platelets, the effectors of clotting, provide another striking case of mechanical responsiveness. High shear stress, as found in injured arteries or stenotic vessels, directly activates platelets. As the platelet mechanosensor, glycoprotein Ib-IX-V complex (GPIb-IX-V) binds von Willebrand factor (vWF) under flow and transduces shear force into intracellular signals [13,14]. In essence, platelets use physical cues to decide when to disband or aggregate: They promote thrombosis when mechanical and biochemical triggers concur, but excessive shear triggers an inbuilt inhibitory feedback to prevent uncontrolled clotting [15].

These examples highlight how blood cells not only transduce forces in the moment but also exhibit mechanical memory and adaptation. The cytoskeleton of a cell can effectively “remember” past mechanical events by undergoing lasting structural changes [16]. Under repeated mechanical stretching by optical tweezers, RBC membrane–cytoskeletal complex undergoes irreversible structural rearrangement [17]. More generally, their response to a given mechanical force is influenced by their prior exposure to similar forces [16]. The vWF binding site and receptor sensitivity are continuously up-regulated by repeated mechanical stimulation, causing the GPIb-IX-V complex of platelets to remain in an activated state [18]. Collectively, these mechanoresponsive behaviors of blood cells—mechanical sensing, learning and memory, decision-making and adaptive evolution—represent what we term “mechanical intelligence”. This blood mechanical intelligence is the integrated capacity of blood cells to sense mechanical forces by mechanosensors and process these signals via encoders to achieve mechanotransduction, thereby enabling learning and memory processes and autonomous response to decide and adapt their behavior accordingly.

**Fig. 1.**
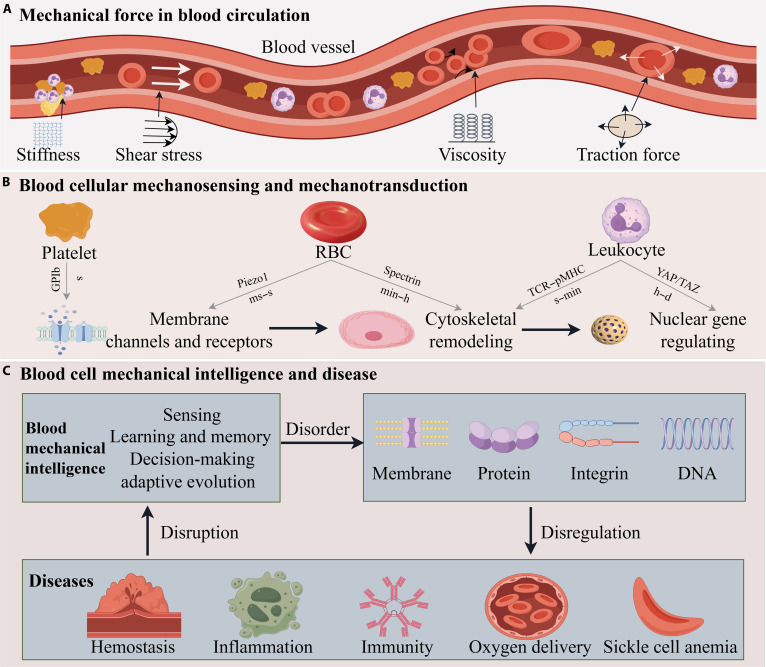
Schematic overview of mechanical forces in blood circulation and blood cellular responses. (A) Fundamental mechanical forces: stiffness, shear stress, viscosity, and traction force. (B) Blood cellular mechanosensing and mechanotransduction pathways across blood cellular components and vasculature, mediated by membrane channels/receptors, cytoskeletal remodeling, and nuclear signaling. (C) Blood cells mechanical intelligence and disease. Blood mechanical intelligence maintains homeostasis, while its dysregulation drives molecular perturbations that trigger disease. These diseases, in turn, disrupt mechanical intelligence via pathological microenvironments.

Viewing blood through the prism of mechanical intelligence has broad implications (Fig. 1). It suggests that blood diseases from sickle cell anemia to atherosclerosis involve failures or alterations in cellular mechanosensing. It also inspires new cross-disciplinary innovations—so-called mechanodiagnostics and mechanotherapies. Measuring the deformability or mechanosignaling of blood cells is emerging as a diagnostic strategy (e.g., microfluidic devices that detect stiff malaria-infected RBCs or hyperreactive platelets [19]. Therapeutically, drugs targeting mechanotransducers such as PIEZO1, or biomaterials that modulate extracellular stiffness, represent novel ways to influence blood cell behavior in disease. In the following sections, we describe the evidence for mechanical sensing, learning and memory, decision-making, and adaptive evolution in blood cells, discuss how these intelligent mechanical behaviors emerge at cellular level, and consider diagnostic applications of blood mechanics and therapeutic opportunities that harness or modulate the mechanical intelligence of blood (see Results and Discussion). By integrating perspectives from medicine and physics, we aim to illuminate a future in which the “smart” properties of blood are leveraged for cutting-edge clinical interventions (see Conclusion). An overview of this review is presented in Boxes 1 to 3.

Box 1. What do we mean by “mechanical intelligence”?

• Scope: Not cognition; an engineering-style control loop in cells: (a) sensors for mechanical sensing (PIEZO1, integrins, GPIb/IX/V, and selectins), (b) encoders (Ca^2+^ pulses, Rho GTPase signaling pathway, and SFK signaling pathways), (c) learning and memory (shape recovery, antigen recognition, and repairing vascular), (d) decision (adhere versus roll, spread versus rest, and contract versus stabilize), and (e) adaptation evolution (cytoskeletal connectivity and chromatin marks).
• Testable predictions: Prior mechanical exposures shift thresholds (e.g., activation, adhesion, and transmigration) in quantifiable ways.
• Falsifiability: If prior exposures do not measurably change thresholds or policies under controlled force histories, the “memory” claim is rejected.

Box 2. Method primer and reporting checklist (mechanodiagnostics)

• Shear profile characteristics, including steady, pulsatile, and oscillatory patterns, along with peak and root mean square values; exposure duration and duty cycle.
• Cell source information, including donor age, sex, and comorbidities.
• Device calibration parameters, such as cantilever spring constants, microchannel dimensions, and flow wverification procedures.
• Measured end points, including deformability indices, stiffness, adhesion lifetime, contractile force, and calcium ion (Ca^2+^) dynamics.
• Interventions involving pharmacologic inhibitors (e.g., PIEZO1 and ROCK blockers), as well as cell-scale and device-scale engineering modifications.

Box 3. Translational readiness levels (TRL-style rubric)

• TRL 2 and 3: Ex vivo mechanophenotyping correlates with disease (e.g., RBC deformability in sickle cell disease and shear-primed platelet assays).
• TRL 4 and 5: Prospective observational studies demonstrate prognostic value (e.g., microfluidic thrombus profiling in hypertension/aging).
• TRL 6 and 7: Interventional studies showing mechanotarget modulation changes clinical end points (e.g., integrin/GPVI axis and PIEZO1 modulators).
• TRL 8 and 9: Device/drug integrated into clinical workflow with demonstrated non-inferiority to standard care plus unique benefit on mechanical end points.

## Results and Discussion

### Mechanical intelligence of blood cells

**Fig. 2.**
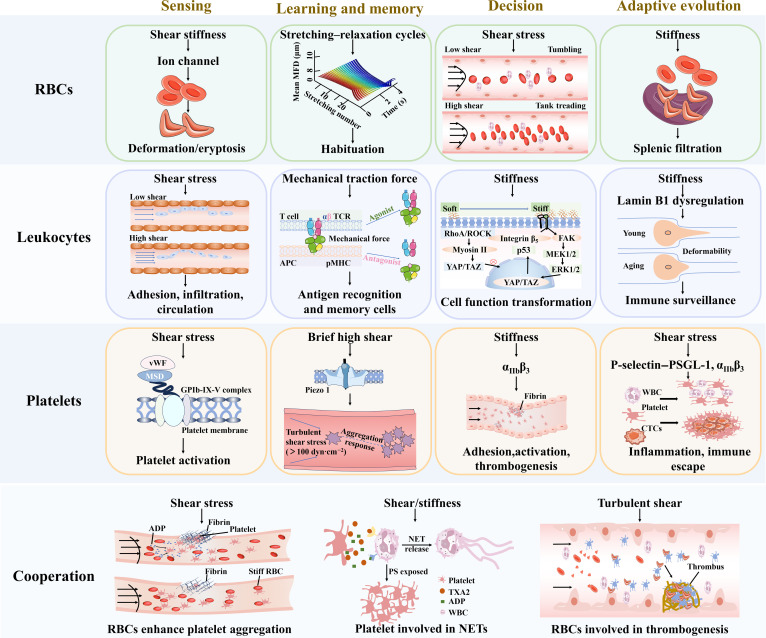
Mechanical intelligence of blood cells. RBCs: Sense shear stress via PIEZO1 channels, triggering Ca^2+^ signals to regulate deformability and oxygen delivery; exhibit mechanical learning under repeated stress: Spectrin cytoskeleton reorganization causes stiffening, and RBCs switch between tumbling and TT modes under varying shear; possess shape memory, reverting to biconcave discs postdeformation; and in microcirculation, make migration decisions via stiffness differences. Leukocytes: Utilize integrins/PIEZO1 to sense mechanical environments: initiate adhesion under low shear stress but deform to avoid adhesion in high shear stress; T cells screen antigens via mechanical force to probe pMHC affinity; make decisions such as proinflammatory/anti-inflammatory in response to mechanical stimuli; and aging increases leukocytes’ stiffness via nuclear lamina alterations, impairing immune surveillance. Platelets: Upon vascular injury, platelet sense shear stress via GPIb-IX complex to be activated; prestimulated platelets exhibit exaggerated aggregability at vascular lesions via PIEZO1; platelets gauge substrate stiffness to decide whether to fully spread, contract, and aggregate; and they also remodel membrane mechanosensors to adapt to aging and regeneration. Cooperation: Mechanical stimulation mediates the interaction among blood cells; under shear stress, RBCs promote platelet aggregation; activated platelets trigger NET release; and sickle RBCs contribute to thrombus formation. FAK, focal adhesion kinase; MFD, maximum feret diameter; PSGL-1, P-selectin glycoprotein ligand-1.

Circulating blood cells display “mechanical intelligence”, i.e., the ability to perceive mechanical forces, encode experience as short- and long-term memory, integrate these cues with biochemical signals, and execute context-appropriate decisions that adapt over time. These mechanical intelligence capabilities allow RBCs, leukocytes, and platelets to independently optimize oxygen transport, immune surveillance, and hemostasis across rapidly fluctuating shear, pressure, and matrix conditions. Meanwhile, they also respond synergistically to mechanical stimuli during certain physiological and pathological processes such as the formation of neutrophil extracellular networks and thrombosis, forming an integrated mechanical regulatory network in the circulatory system. Below, we synthesize the core behaviors, including mechanosensing, mechanical learning and memory, decision-making, and adaptive evolution, for each lineage, emphasizing quantitative thresholds where available, and further elaborate on the collective mechanical behaviors arising from intercellular cooperation in the bloodstream (Fig. 2).

#### Red blood cells

The ability of blood cells to sense mechanical forces in their microenvironment, including shear stress, stiffness, viscosity, and traction force, is termed blood mechanosensing. RBCs possess a sophisticated form of mechanosensing intelligence, allowing them to detect and transduce mechanical forces into biochemical activity. PIEZO1 is a crucial mechanical sensor on RBCs that converts mechanical stimuli into biological signals. It not only regulates other proteins synthesis but also controls iron metabolism, linking to erythropoiesis. Its mutations can also delay reticulocyte maturation, affecting glycolytic metabolism and oxygen affinity. Membrane stretch or shear activates PIEZO1, thereby modulating ion flux to enable RBCs to continuously adjust membrane viscosity and elasticity, which minimizes energy dissipation across shear regimes and contributes to dehydration control [20].

Habituation is a form of cellular learning [21]. Both in vivo circulation and in vitro optical-tweezers-induced stretching experiments demonstrate that repeated deformation induces mechanical habituation in RBCs, indicating that RBCs possess mechanical learning and memory capabilities. This process involves hemoglobin–actin network remodeling (mechanical protection), leading to progressive reduction in deformability, increased stiffness, and accelerated accumulation of membrane damage alongside cytoskeletal remodeling. This adaptive strategy delays fatigue [17,21,22]. “Mechanical memory” is claimed to retain cellular information from past mechanical microenvironments and influence subsequent cell fate [23]. RBCs “remember” their biconcave geometry. When exposed to shear flow that induces tank treading (TT) or elongation and subsequently returned to static condition, RBC membranes tend to drift back to their original configuration. In go–stop shear experiments, membrane-attached fiducial markers returned to their initial positions even after hours of TT, and RBCs recovered their discoid shape at both room temperature and 37 °C, indicating the evidence of a persistent internal stress topology within the membrane skeleton [24]. Such shape memory ensures that RBCs can repeatedly squeeze through tiny capillaries and recover their optimal discoid shape for efficient gas exchange. To sum up, blood cells can “remember” past mechanical stimuli through “learning” to remodel encoders following repeated mechanical sensing—a phenomenon that we term blood mechanical learning and memory.

The ability of blood cells to make cellular fate decisions based on mechanical sensing and mechanotransduction is termed blood mechanical decision-making. RBCs execute a form of mechanical decision-making by switching between distinct dynamic modes in response to hydrodynamic conditions. Below a shear threshold dependent on cytoskeletal elasticity and cytoplasmic viscosity, RBCs tumble; beyond it, they transition to TT [24]. TT aligns cells with flow and reduces asymmetric stress at high shear, whereas tumbling prevents prolonged deformation at low shear, representing an energy-saving, mechanical decision-making choice [25]. Mechanical heterogeneity decides how RBCs respond to hydrodynamic forces. Soft healthy RBCs navigate high shear arterioles via TT, whereas stiff pathological RBCs marginate toward vessel walls, predisposing to microvascular occlusions, as confirmed by simulations and in vivo observations (e.g., in sickle cell disease) [26].

#### Leukocytes

Leukocytes, which can be classified into 5 major categories (neutrophils, eosinophils, basophils, lymphocytes, and monocytes), play essential roles in immune defense. They can integrate shear forces and substrate stiffness via selectins, integrins, and cytoskeletal architecture to navigate the vasculature. Rolling and adhesion are optimal at ~0.1 to 5.0 dynˑcm^-2^ of shear stress, mediated by selectin catch bonds that lengthen bond lifetime under modest tension. However, beyond 10 dynˑcm^−2^, adhesion probability declines by 90%, as bonds transition to slip behavior, and cells undergo morphological changes to a teardrop shape before detachment [27,28].

Leukocytes can “remember” prior mechanical exposures, which can alter their responsiveness upon subsequent challenges. Brief subthreshold shear primes neutrophils for enhanced neutrophil extracellular traposis upon a second hit, consistent with a short-term mechanical memory that lowers activation thresholds [29]. Other clear examples are the behavior of lymphocytes during antigen recognition. Liu et al. [30] investigated the effects of mechanical forces on TCR–peptide major histocompatibility complex (TCR–pMHC) interactions. T cell contact with antigen-presenting cells (APCs) induces cytoskeletal contraction, generating physiological forces of 5 to 10 pN. These forces regulate TCR–pMHC binding patterns and determine whether TCR undergoes conformational changes to expose immunoreceptor tyrosine-based activation motif (ITAM) for T cell activation. Similarly, following antigen binding, the traction force on B cells increases rapidly, reaching a peak within 10 to 15 min. This is followed by a plateau phase (lasting 20 to 30 min) and then gradually decreases as antigen internalization is completed. Under high-affinity antigen stimulation, the peak traction force (35 to 40 pN) is significantly higher than that induced by low-affinity antigens (15 to 20 pN). When traction falls below 25 pN, antigen–B cell receptor (BCR) binding is easily disrupted, preventing effective activation. Memory B cells exhibit a slightly lower peak traction force (25 to 30 pN) than naïve B cells (30 to 40 pN) but reach peak force more rapidly (5 to 8 min), consistent with the “rapid response” functional characteristic of memory B cells [31]. These memories are plausibly stored in cytoskeletal architecture and mechanosignaling states that persist after stimulus removal.

Leukocytes weigh mechanical and chemical cues to choose between adhesion versus deadhesion, circulation versus extravasation, phagocytosis versus neutrophil extracellular trap (NET) release, and proinflammatory versus anti-inflammatory programs. During diapedesis, increasing shear initially stabilizes rolling through catch bonds; once integrins engage, shear promotes cell spreading and migration through endothelial junctions. However, excessively high shear stress defers transmigration to regions of lower stress, such as postcapillary venules [28,32]. Matrix mechanics polarize macrophages: Stiff (~100 kPa) substrates bias toward M1 via PIEZO1–Yes-associated protein (YAP) signaling, whereas soft (~1 to 5 kPa) matrices favor M2 phenotypes [33]. Moreover, endothelial PIEZO1 is required cell-extrinsically for efficient leukocyte diapedesis [34].

Aging imposes a robust biomechanical signature on immune cells. CD4^+^/CD8^+^ T cell elastic modulus increases from ~0.5 to 1.0 kPa to ~2.0 to 4.0 kPa, linked to nuclear lamina disorganization (e.g., Lamin B1 loss), reduced deformability, and impaired interstitial migration and capillary transmigration [35,36]. In addition, age-associated dysregulation of integrin and actin dynamics further degrades diapedesis efficiency in neutrophils and monocytes [37]. Thus, immunosenescence is characterized by a mechanophenotypic drift that impairs immune surveillance: As leukocytes accumulate mechanical and oxidative damage over a lifetime, they undergo adaptive changes, such as increased stiffness and reduced motility, which further contribute to the deterioration of immune function. In all, blood cells can adapt their behaviors based on the past mechanical responses by remodeling cytoskeletal connectivity or gene programs—a phenomenon that we term blood mechanical adaptive evolution.

#### Platelets

Mechanical cues regulate platelet output by mechanosensing. In megakaryocytes (MKs), arterial-like shear stress acutely accelerates proplatelet formation, with 30 to 45% of MKs releasing platelets within ~20 min under flow, and turbulence boosts ex vivo biogenesis to clinical scale [38]. PIEZO1 acts as a negative mechanical regulator of MK maturation; its activation suppresses polyploidization and platelet yield, whereas deletion increases platelet counts, establishing a feedback control on circulating numbers [39]. Matrix stiffness also tunes thrombopoiesis: Stiff substrates restrain proplatelet formation via α_IIb_β_3_-dependent spreading/contractility, while transient receptor potential vanilloid 4 activation on soft matrices promotes calcium (Ca^2+^) signaling and platelet production [40].

Once in the bloodstream, platelets are constantly monitoring the mechanical environment for signs of vessel injury. At a wound site, when subendothelial collagen and vWF are exposed, platelets rapidly sense the abrupt change in mechanical conditions: from a smooth endothelial surface to a rough, adhesive, and sometimes highly strained matrix [15]. High shear stress unfolds vWF’s A1 domain (≥500 to 1,000 s^−1^) to engage platelet’s GPIbα, initiating tethering and a GPIb-driven Ca^2+^ spike that activates α_IIb_β_3_; parallel GPVI–collagen binding senses the rigid collagen matrix to consolidate activation [41–43]. These mechanical signals are converted into biochemical signals within platelets, activating them to participate in hemostasis. Furthermore, GPVI deficiency (e.g., patients with Ehlers–Danlos syndrome) underscores this mechanosensing requirement, manifesting as bleeding due to impaired collagen responses [44].

Platelets also exhibit mechanical learning and memory. Traditional views hold that platelets primarily undergo passive adhesion and aggregation at sites of vascular injury to form thrombi for hemostasis. Nicolai et al. [45] observed that platelets actively monitor blood vessels through chemotaxis (migration along a gradient of matrix molecules), rapidly aggregating along collagen gradients exposed at sites of inflammation/infection, ultimately repairing the vascular barrier, limiting inflammatory spread, and enhancing infection defense—independently of the classical coagulation pathway. In addition, brief exposure to high shear stress (e.g., ~60 dynˑcm^−2^ for ~40 s) primes platelets to respond to subsequent low shear stress (~1 dynˑcm^−2^) with markedly enhanced aggregation (~20-fold) compared to naïve platelets that never suffer the high shear. This priming requires Ca^2+^ signaling generated via PIEZO1 and other mechanosensor and can be attenuated pharmacologically [18,46]. This mechanical learning and memory is beneficial: After platelets pass through high shear stenoses, they will more readily form a hemostatic plug downstream if needed. Conversely, sustained extreme shear stress (e.g., in patients on cardiopulmonary bypass or with mechanical heart valves) causes platelet depletion, degranulation, and functional exhaustion, producing a paradoxical thrombosis/bleeding phenotype clinically [47].

Platelets sense mechanical force (e.g., shear stress and substrate stiffness) to decide whether to fully spread, contract, and aggregate. Under normal physiological shear stress conditions, platelets appear as smooth, disc-shaped structures. When exposed to high shear stress environments, such as narrowed blood vessels, platelets rapidly respond by first extending filamentous pseudopodia. This increases their contact surface area with the vessel wall or other platelets, facilitating adhesion and aggregation. As shear stress persists, platelets undergo further deformation, producing more pseudopodia that interconnect to form a network structure, thereby promoting the formation of platelet aggregation [48]. In addition, on rigid fibrin (such as a calcified plaque or a taut fibrin mesh), they display greater α_IIb_β_3_ activation, α-granule secretion, and rapid conversion to a procoagulant state through Rho-associated protein kinase (ROCK)–myosin light chain pathway-driven traction [49]. Moreover, filamin A (FLIN A) links α_IIb_β_3_ to the cytoskeleton, forming a mechanical positive feedback required for force generation and clot retraction [50]. Therefore, platelet involvement in stabilizing hemostatic thrombi constitutes a force-threshold-regulated decision-making mechanism.

Platelets also use mechanical cues to adapt evolution in immunity and aging processes [51,52]. Under high shear, platelets aggregate with circulating tumor cells (CTCs) and leukocytes via α_IIb_β_3_ or P-selectin, shielding CTCs and modulating inflammation [53–55]. In sepsis or arterial injury, platelet–neutrophil interactions enhanced by shear stress stimulation promote excessive NET formation, which intertwines with fibrin to cause microvascular occlusion [50]. In addition, local collagen stiffening can promote platelet-derived microparticles, amplifying inflammatory signaling [56]. During aging, loss of GPIbα sialic acid marks platelets for Ashwell–Morell receptor-mediated clearance and triggers compensatory thrombopoietin production, thereby embedding life cycle of platelet within a mechanoendocrine feedback loop [57–60].

#### Collective mechanical intelligence in the bloodstream

These mechanical behaviors often occur in cooperation among different cells, rather than in isolation within a single cell. Under in vitro flow conditions, RBCs can enhance platelet aggregation on collagen surfaces. In mouse models, transfused RBCs also improved hemostasis with severe pancytopenia by enhancing fibrin formation. Similarly, increasing RBC membrane stiffness interferes with shear-induced platelet aggregation, while adenosine diphosphate (ADP) released by RBCs under high shear stress promotes platelet activation. Accordingly, altering the deformability of RBC membranes affects their capacity to support platelet function [61]. NETs are reticular structures released by activated neutrophils, containing components such as DNA, histones, and antimicrobial proteins. NETs are widely present in inflammation, infection, and vascular diseases. They can directly damage vascular endothelial cells, promote thrombosis, and trigger vascular inflammatory responses, making them one of the key pathological mechanisms underlying vascular complications such as atherosclerosis, thromboembolism, and ischemic stroke. Platelets can activate neutrophils by releasing bioactive substances (such as ADP and thromboxane A2 [TXA2]), thereby inducing NET release. Concurrently, direct platelet–neutrophil interactions also enhance NET production. Phosphatidylserine (PS) exposed on the NET surface provides a platform for platelet activation, promoting platelet aggregation and clotting factor assembly. This accelerates thrombin generation and fibrin deposition, ultimately forming a thrombus [29,62]. Pathological force fields can invert these synergies: Turbulent shear fragments RBCs and hyperactivates platelets (hemolysis–thrombosis), while rigid sickled RBCs marginate, interact aberrantly with endothelium and platelets, and initiate microthrombi [26]. Understanding these multicell, force-coupled loops clarifies how the vasculature maintains homeostasis and how their dysregulation drives inflammation and thrombosis.

### The mechanism of mechanical intelligence formation of blood cells

Blood cells sense force stimuli through sensors such as PIEZO1, integrins, GPIb/IX/V, and selectins and transduce mechanical signals by encoders (Ca^2+^ pulses, Rho guanosine triphosphatase [Rho GTPase] signaling pathway, and Src family kinase [SFK] signaling pathways). During these processes, mechanical intelligence emerges from 3 nested layers of information processing: membrane mechanoreception, cytoskeletal force transduction, and nuclear mechanotransduction (in nucleated cells). These layers encode short-term memory (ion fluxes and posttranslational signaling), intermediate structural memory (cytoskeletal remodeling), and long-term memory (epigenetic/gene expression changes), thereby enabling blood cells to make decisions, and undergo adaptive evolution (Table 1).

**Table 1.** Comparative mechanisms of blood cell mechanical memory

Cell type	Memory type	Sensors	Encoders	Timescale	Reversibility	Refs.
RBC	Mechanical fatigue and deformation memory	PIEZO1	Ca^2+^ signaling pathway	Long term (hours to days)	Partially reversible	[63,74,75]
	Shear adaptation memory	Spectrin, actin	Cytoskeletal dynamic reorganization	Short term (minutes to hours)	Reversible	[80]
	Aging/disease-related stiffening memory	Primarily structural changes	Membrane protein cross-linking; lipid oxidation, skeleton reorganization	Long term (days to weeks)	Irreversible	[22,80]
Leukocyte	Haptotaxis and migration memory	Integrins, selectins	Rho GTPase signaling pathway; “catch-bond” kinetics	Short term (minutes to hours)	Reversible	[68,73]
	Stiffness sensing and polarization memory	PIEZO1, integrins, TLR4, YAP/TAZ	Ca^2+^ signaling pathway	Medium to long term (hours to days)	Partially reversible	[33,64,76,82]
			Integrin–ROCK–myosin II axis			
	Force history-dependent epigenetic memory	Integrins/PIEZO1	Epigenetic reprogramming	Long term (days to weeks)	Partially reversible	[34,81]
Platelet	Shear activation memory	GPIb-IX-V, PIEZO1, P2X1	Ca^2+^ signaling pathway; GPIb-IX-V complex combined with vWF; PIEZO1/P2X1 “mechanosome” sense extensional strain	Short term (seconds to minutes)	Partially reversible	[66,70,71]
	Stiffness sensing and spreading/aggregation memory	α_IIb_β_3_, FLIN A	Coordinated Rho GTPase and SFK signaling; Rac1/Cdc42–WASp/WAVE–Arp 2/3 pathway; SFK–PI3K–AKT pathway; Rho GTPase signaling pathway	Short to medium term (minutes to hours)	Partially reversible	[43,49,72,77,78]
	Age-related proinflammatory functional shift	Microenvironment and systemic signals	Signaling pathway bias and epigenetic modifications	Long term (days to weeks)	Irreversible	[57]

#### Sensors for mechanical sensing

Mechanical cues in the vascular microenvironment critically regulate blood cell functions, and RBCs, leukocytes (neutrophils and lymphocytes) and platelets use distinct yet conserved mechanosensors—including ion channels, integrins, and cytoskeleton-associated proteins—to perceive and to convert force into biochemical signals.

##### Ion-channel-type mechanosensors

Ion-channel-type mechanosensors are important for blood cells to sense mechanical signals. Among them, PIEZO1 is the most well studied and core member, while ligand-gated P2X receptors (P2X1) also participate in the mechanical signal transduction process of platelets.

In RBCs, PIEZO1 serves as the core mechanosensitive receptor and a key mediator for sensing cyclic shear stress. When RBCs pass through narrowed capillaries or are in a high shear stress environment, the increase in membrane tension triggers conformational changes in PIEZO1, mediating Ca^2+^ influx to regulate cell functional states and help cells adapt to the environment [63]. In leukocytes, PIEZO1 is the most clearly studied ion-channel-type mechanosensitive receptor. High-intensity membrane tension endured by neutrophils during transendothelial migration directly activates PIEZO1 channels, mediating Ca^2+^ influx, which ultimately potentiates reactive oxygen species (ROS) generation and enhances bacterial killing capacity [64]. Meanwhile, PIEZO1 can transduce hemodynamic shear stress into Ca^2+^ signals, driving neutrophils to differentiate into a proangiogenic phenotype and regulating pulmonary vascular homeostasis and regeneration [65]. PIEZO1 of platelets also senses hemodynamic shear stress and extensional strain. Under high shear conditions (e.g., at arterial stenosis where the shear rate reaches 12,000 s^−1^), its activation can mediate Ca^2+^ influx [66]. Under supraphysiological hemodynamic gradients, PIEZO1 acts as an extensional strain sensor, forming a “mechanosome” complex with P2X1 channels to convert mechanical strain signals into Ca^2+^ transients, activating platelets, and putting them in a “sensitized” state under extreme flow conditions [66].

##### G-protein-coupled receptor-type mechanosensors

G-protein-coupled receptors are another important class of mechanosensors that indirectly respond to fluid shear stress by regulating their constitutive activity, playing a unique role in the microcirculation adaptation of neutrophils. Among them, the formyl peptide receptor (FPR) is a typical representative of this class of receptors. When neutrophils are exposed to intravascular shear stress (0.5 to 5 dynˑcm^−2^), the constitutive activity of FPR decreases, which, in turn, inhibits Ras-related C3 botulinum toxin substrate 1 guanosine triphosphatase function, induces pseudopod retraction, and reduces microcirculatory retention. In contrast, undifferentiated HL60 cells (neutrophil precursors) lack FPR expression, resulting in minimal pseudopod formation and no response to fluid shear stress [67].

##### Adhesion receptor family mechanosensors

Various molecules in the adhesion receptor family can act as mechanosensors, which transduce tensile signals at the adhesion interface through dynamic interactions with the extracellular matrix, regulating the adhesion, migration, and other functions of blood cells. They mainly include the integrin family (α_m_β_2_/CD11b and α_IIb_β_3_) and GPIb-IX-V complex.

In leukocytes, integrin α_m_β_2_/CD11b is an important mechanosensitive receptor of the adhesion receptor family. The β_2_ integrin/intercellular adhesion molecule-1 (ICAM-1)–ROCK–myosin II axis mediated by it can convert matrix stiffness into RhoA-dependent contractile force, directly promoting the transendothelial migration of leukocytes [68]. In platelets, the GPIb-IX-V complex is the core mechanosensitive receptor responding to high shear stress, which mainly senses and transduces mechanical signals through its interaction with vWF [69]. Specifically, high shear force can unfold the vWF A1 domain, promoting its binding to GPIbα. Then, the tensile force on GPIbα causes it to expose the juxtamembrane mechanosensory domain (MSD), triggering a strong Ca^2+^ spike, driving α_IIb_β_3_ activation, and ultimately achieving firm adhesion and aggregation of platelets under flow conditions [70,71]. Integrin α_IIb_β_3_, as the main mechanosensitive adhesion receptor in platelets, senses mechanical forces generated by cell–cell and cell–matrix interactions through bidirectional signaling to regulate platelet aggregation [72].

##### Antigen-specific immune-receptor-type mechanosensors

BCR and TCR, as antigen-specific immune receptors, have intrinsic mechanosensitive properties [73]. During interaction with APCs, BCR and TCR can sense interfacial mechanical forces to regulate the intensity of signal activation. This mechanical regulation converts mechanical signals into biochemical signals through cytoskeleton-derived forces, thereby regulating the activation and proliferation of lymphocytes [73].

#### Encoders for mechanotransduction

##### Ca^2+^-dependent signaling pathway

The Ca^2+^-dependent signaling pathway is a core pathway for blood cells to transduce mechanical signals, with PIEZO1 channels often serving as the upstream mechanical sensor to initiate Ca^2+^ influx, which further regulates downstream effector molecules and functional responses.

RBCs can form “mechanical learning and memory” through Ca^2+^-dependent signaling pathway. Calcium ions mediate the volume homeostasis of human erythrocytes in circulation. Deformation of erythrocytes traversing capillaries transiently activates mechanosensitive PIEZO1 channels, allowing Ca^2+^ influx against its steep inward gradient. This temporarily overrides the calcium pump and elevates [Ca^2+^]i [63]. This Ca^2+^ signal also activate downstream effectors including the Gardos channel (KCa3.1) and scramblase: KCa3.1-mediated K^+^ efflux induces water loss to reduce RBC volume and enhance deformability, facilitating passage through microvasculature, while scramblase activation promotes PS exposure on the cell membrane [74]. In addition, PIEZO1-dependent Ca^2+^ influx regulates mechanotransductive adenosine triphosphate release from RBCs, which modulates vascular tone and endothelial function—an effect blunted by PIEZO1 inhibitors such as grammostola spatulata mechanotoxin 4 (GsMTx4) or ruthenium red [75].

In leukocytes, Ca^2+^-dependent signaling is crucial for immune function execution. During transendothelial migration or tissue infiltration, neutrophils sense shear stress or compressive forces via PIEZO1; sustained compressive forces during capillary traversal activate membrane-localized PIEZO1, mediating transient Ca^2+^ influx that drives up-regulation of proangiogenic genes (e.g., vascular endothelial growth factor A) and inflammation-related genes [65]. Meanwhile, PIEZO1 activation by high-intensity membrane tension during neutrophil transendothelial migration mediates Ca^2+^ influx, potentiating ROS generation and enhancing bacterial killing capacity [64]. For macrophages, PIEZO1-mediated Ca^2+^ signaling couples to calcium/calmodulin-dependent protein kinase II–hypoxia-inducible factor-1α (CaMKII–HIF-1α) to promote glycolysis and modulates M1/M2 polarization via actin feedback [76]. In addition, B cells sense mechanical traction through BCR-membrane antigen binding, triggering a Ca^2+^ effector-involved signaling cascade that induces BCR microclustering, antigen extraction, and full B cell activation [31]; TCR binding to pMHC activates Ca^2+^-related mechanotransduction, amplifying TCR signaling and inducing T cell activation [11].

In platelets, Ca^2+^ signaling participates in the “sliding–locking” positive feedback during mechanotransduction. Under high shear conditions, PIEZO1 activation mediates Ca^2+^ influx [66]; subsequently, Ca^2+^-dependent myosin II contraction forms stress fibers, while talin/kindlin 3 stretching reinforces α_IIb_β_3_ activation [72]. Moreover, tensile force on GPIbα exposes the juxtamembrane MSD, triggering a strong Ca^2+^ spike that drives α_IIb_β_3_ activation and firm platelet adhesion and aggregation [70,71].

##### Rho GTPase signaling pathway

The Rho GTPase signaling pathway mainly mediates cytoskeletal remodeling, cell adhesion, and migration in blood cells by transducing mechanical signals from adhesion receptors or membrane mechanosensors.

In leukocytes, this pathway is critical for neutrophil recruitment and migration. Integrins such as β_2_ perceive adhesive tension at the leukocyte–endothelium interface, activating Rho GTPase pathways to reinforce cytoskeletal remodeling and stable adhesion—key processes for neutrophil recruitment to inflammation sites [68]. During neutrophil diapedesis, Rho-GTPase-driven actin polymerization generates lamellipodia and invasive protrusions; inhibition of nonmuscle myosin II (a principal tension motor) reduces migration efficiency by >70%, highlighting the centrality of actomyosin contractility mediated by this pathway. In addition, the β_2_ integrin/ICAM-1–ROCK–myosin II axis in leukocytes (a branch of Rho GTPase signaling) converts matrix stiffness into RhoA-dependent contractile force, directly promoting transendothelial migration [68].

In platelets, Rho-GTPase-related molecules participate in actin remodeling during mechanotransduction. Triggered by integrin α_IIb_β_3_-mediated outside-in signaling, platelets activate the Ras-related C3 botulinum toxin substrate 1/cell division control protein 42 homolog/Wiskott–Aldrich syndrome protein/WASp-family verprolin-homologous protein (Rac1/Cdc42–WASp/WAVE) axis (members of the Rho GTPase family) to drive the actin-related protein 2/3 (Arp2/3) complex in assembling branched actin networks at the periphery, promoting lamellipodia formation and haptotactic migration along substrate-bound gradients [77].

##### SFK signaling pathways

SFK-related signaling pathways play important roles in platelet activation, leukocyte immune synapse formation, and other mechanical signal transduction processes, often converging with other pathways to enhance functional responses.

In platelets, high shear stress induces combined GPIbα and GPVI signaling, which engages SFK and phosphatidylinositol 3-kinase protein kinase B (PI3K–Akt) pathways. These pathways converge with soluble agonists to fully activate α_IIb_β_3_ and drive irreversible aggregation; subthreshold shear biases toward noncommitment, limiting inappropriate thrombosis [43,78]. In addition, high shear stress on the platelet endothelial cell adhesion molecule 1/vascular endothelial cadherin/vascular endothelial growth factor receptor 2 complex leads to immunoreceptor tyrosine-based inhibitory motif (ITIM) phosphorylation and ITIM signaling, regulating platelet alignment, nitric oxide production, and inflammatory gene expression [79]. The spleen tyrosine kinase–PI3K–WAVE2–Arp2/3 cascade (linked to SFK signaling) mediates rapid branched F-actin assembly in platelets within seconds of mechanical stimulation [77].

In leukocytes, SFK signaling is involved in T and B cell activation. TCR binding to pMHC triggers activation of Src family kinases (e.g., Lck and Fyn), which phosphorylate TCR–CD3 ITAMs, recruit adaptor proteins (zeta-chain associated protein 70 kDa and linker for activation of T cells), and drive cytoskeletal remodeling via actin regulators (WASp and Arp2/3), amplifying TCR signaling [11]. Similarly, B cells sense mechanical traction through BCR–membrane antigen binding, initiating a signaling cascade of Src family kinases and cytoskeletal remodelers that induces BCR microclustering and full activation [73]. For macrophages, PIEZO1 can act as a Toll-like receptor 4 (TLR4) co-receptor, enhancing phagocytosis via SFK-related signaling [76].

##### Epigenetic and metabolic reprogramming

In anucleate RBCs, mechanical memory is encoded exclusively in material properties: Repeated optical-tweezers-induced stretching progressively stiffens the spectrin network (mechanoprotection), diminishes shape recovery (mechanical fatigue), and primes RBCs for splenic sequestration and elimination [22,80]. Evolution-refined spectrin–ankyrin cross-linking balances elasticity with resilience, enabling repeated passage through 2- to 3-μm slits and rapid shape recovery [80], while environmental shear stress promotes traits enhancing deformability in high shear arterial beds and antiaggregation in low shear environment [61].

In leukocytes, force history reprograms transcription via epigenetic modifications. Monocytes repeatedly traversing basement membranes acquire histone H3 lysine 27 acetylation activation marks on latent enhancers of migration-related genes (e.g., matrix metallopeptidase 9 and integrin subunit β2), enhancing gene expression and increasing migration efficiency by 3.5-fold (third versus first crossing); inhibiting these enhancers or blocking histone deacetylation erases this migration memory [34,81]. For macrophages, PIEZO1-mediated CaMKII–HIF-1α signaling promotes glycolysis (metabolic reprogramming), regulating M1/M2 polarization and linking mechanical cues to tissue repair [33,82].

### Mechanodiagnostics and mechanotherapy: Harnessing blood cell mechanical intelligence

**Fig. 3.**
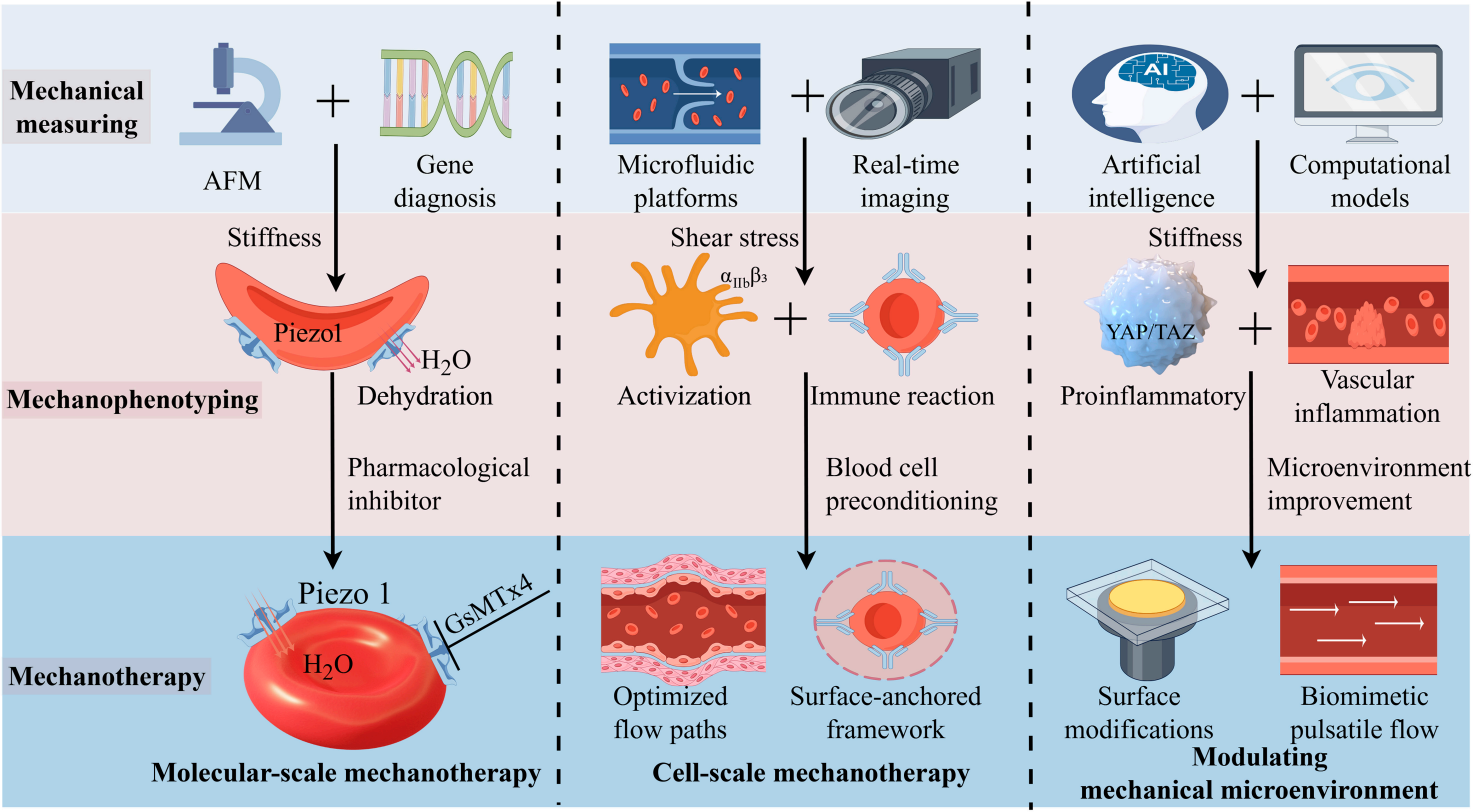
Integrated mechanodiagnostics and mechanotherapy strategies targeting blood cells mechanical intelligence. AFM is utilized to quantify cellular stiffness through mechanical measuring, mechanophenotyping the PIEZO1 mechanosensitive ion channel that regulates cellular hydration (H_2_O), where pharmacological inhibitors correct dehydration pathologies by blocking aberrant PIEZO1 activity. Microfluidic platforms enable shear-dependent analysis and real-time deformability imaging, with optimized flow paths to reduce platelet activation or engineered RBCs to avoid immune reactions. Artificial-intelligence-enhanced computational models predict abnormal cell and vascular disease, collectively creating mechanically biocompatible microenvironments to advance mechanotherapy.

Blood cells exhibit intrinsic “mechanical intelligence”—the ability to convert mechanical forces into signals and behaviors, such as mechanosensing, learning and memory, and decision-making, which underpins novel diagnostic biomarkers and druggable nodes. We frame translation as measure, mechanophenotype, and modulate: (a) measure mechanical environments, single-cell mechanics, and mechanosensing and mechanotransduction of molecular level with calibrated platforms; (b) mechanophenotype mechanical environments, single-cell mechanical properties, and mechanosensing and mechanotransduction processes in relation to clinical states; (c) modulate aberrant mechanosensing and mechanotransduction with targeted molecular-scale therapies and cell-scale or device-scale engineering (Fig. 3).

#### Measuring mechanical properties for mechanodiagnostics

The technologies to measure mechanical properties of blood cells for early disease screening, diagnosis, condition monitoring, and prognosis assessment, we call it mechanodiagnostics. Accurate quantification is the foundational step, relying on a suite of biophysical tools. Techniques such as atomic force microscopy (AFM), micropipette aspiration, and optical tweezers provide high-resolution, single-cell data on stiffness, cortical tension, and deformability under static or quasi-static conditions [83,84]. These methods are essential for establishing baseline mechanical constants. Concurrently, dynamic, population-level measurements are crucial. Microfluidic platforms subject cells to well-defined fluid shear stresses and capillary-scale constrictions, generating high-throughput data on deformation kinetics and recovery times that closely mimic in vivo hemodynamics [85–87]. In particular, the emergence of some faithful models that can accurately distinguish pathological blood cells has brought significant advantages to mechanodiagnostics in the treatment of blood diseases [88].

The evolution of these tools is marked by increasing throughput and biological integration. Early single-cell methods were precise but low-throughput. The development of high-throughput deformability cytometry, including real-time deformability cytometry, represented a significant leap [19]. Recent integration of such mechanical screening with omics technologies, such as single-cell RNA sequencing, allows for direct correlation of mechanical states with molecular profiles, enabling the discovery of genetic regulators that set a cell’s mechanical “set point” [19]. This convergence is essential for moving beyond descriptive phenomenology toward mechanistic discovery.

#### Mechanophenotyping mechanical profiles of disease states

The mechanical characteristics of different cell states are what we term a mechanophenotype. The critical next step is mechanophenotyping—the systematic association of mechanical signatures with specific clinical states to identify “mechanobiomarkers” and reveal “mechanopathologies”. For example, the pronounced loss of deformability in plasmodium-infected or sickle erythrocytes is a direct driver of microvascular occlusion, serving as a quantifiable metric of disease severity [85]. In leukocytes, lymphocyte stiffening is documented in severe COVID-19, while altered monocyte mechanics correlate with inflammatory and neoplastic conditions, offering a multidimensional view of immune dysfunction [89].

These abnormal cellular mechanopathologies also trigger blood function abnormalities. In sickle cell disease, erythrocyte rigidity directly causes vaso-occlusion, and the resulting thrombi exhibit high stiffness but low fracture toughness, predisposing to embolization [85]. In platelets, mutations in nonmuscle myosin IIA (MYH9-related disorders) impair force generation, leading to deficient clot retraction—a pure mechanical dysfunction [90]. Conversely, in acute myeloid leukemia, malignant cells can actively soften to evade cytotoxic lymphocyte attack at the immune synapse [89]. These examples demonstrate how discrete mechanical failures drive diverse clinical phenotypes through the common lens of dysregulated cellular mechanobiology.

#### Modulating mechanosensing and mechanotransduction for mechanotherapy

Physical or pharmacological therapeutic approaches targeting blood system mechanical characteristics—a strategy that we term blood mechanotherapy. At the molecular level, “mechanodrugs” such as PIEZO1 blocker GsMTx4 (for hereditary xerocytosis), anti-GPIbα fragment, or CD31 agonist peptides (for vascular inflammation) directly target aberrant force-sensing apparatuses [91–93]. Similarly, inhibiting YAP/transcriptional coactivator with PDZ-binding motif (TAZ) signaling or using lesion-specific nanoparticle delivery (e.g., verteporfin) can counteract proinflammatory mechanotransduction [94]. These interventions aim to reset aberrant activation thresholds for adhesion or aggregation.

Modulation extends to cellular engineering and device design. Targeting the extracellular mechanical microenvironment can modulate cellular behaviors [95,96]. Cells can be mechanically “armed” or “trained”. For instance, surface-anchored polymer frameworks create stealth RBCs that evade immune clearance without compromising deformability, and ex vivo mechanical preconditioning can enhance cell resilience [97,98]. Engineered chimeric antigen receptor T cells expressing extracellular-matrix-degrading enzymes exemplify overcoming biomechanical barriers in solid tumors. On a macroscale, optimizing the hemocompatibility of blood-contacting devices is crucial. This involves introducing biomimetic pulsatility in extracorporeal membrane oxygenation (ECMO), applying endothelium-inspired antithrombotic coatings to prevent factor XII (FXII) activation and using computational fluid dynamics to design geometries that minimize shear stress hotspots in ventricular assist devices (VADs) [99–102].

The key mechanical parameters (such as shear stress and stiffness), recommended reporting standards, and available public resources for blood cell mechanobiology experiments were also summarized in Table 2. Collectively, these strategies aim to restore physiological mechanical homeostasis. Key near term goals need be achieved. First, we need to establish mechanodiagnostic platforms. For example, adding monitoring modules for mechanical properties to existing blood diagnostic equipment not only reduces costs of instrument development but also facilitates clinical translation. Second, we need to standardize mechanical readouts across platforms, embed mechanical end points in clinical samples (e.g., RBC relaxation time and platelet aggregate force under different physiological and pathological conditions), and developing companion diagnostics that match patients to mechanotransduction-targeted therapies.

**Table 2.** Mechanical properties and experimental characterization of blood cells

Cell types	Force				Exposure duration	Simulation and analysis tools	Standard microfluidic designs	Refs.
	Shear stress		Stiffness ranges					
	Physiological	Pathological	Physiological	Pathological				
**RBC**	18–140 dynˑcm^−2^	140-1,750 dynˑcm^−2^, ADP release; >1,750 dynˑcm^−2^, mechanical damage	~3.8 μN·m^−1^; Young’s modulus: 180 Pa in buffer, 100 Pa in plasma (AFM); morphology-dependent stiffness: biconcave, 3.37 ± 0.40 μN·m^−1^; spherical, 3.48 ± 0.23 μN·m^−1^; crenelated, 3.80 ± 0.22 μN·m^−1^	Sickle cell trait: ~4.4 μN·m^−1^	Seconds (shape recovery in flow); minutes (single-cell indentation); hours to days (fatigue or storage studies).	Computational fluid dynamics; hertz model (indentation); Kelvin–Voigt model; membrane spectrin–network models.	Gas-permeable chips for O_2_ control; constriction arrays (splenic slit mimic); coated channels for adhesion assays; modular optical tweezers setups for noncontact manipulation.	[61,107–112]
**Leukocyte**	~0.01–0.15 dynˑcm^−2^;	100 dynˑcm^−2^ deadhesion.	B lymphocytes: resting, 104.28 ± 21.77 pN; T lymphocytes: naïve CD4^+^ T cells, 4–6-fold stiffer than effector CD4^+^ T cells; effector CD4^+^ T cells, softer, lower modulus	B lymphocytes: pokeweed-mitogen-activated, 304.16 ± 60.30 pN; *Staphylococcus aureus* cowan strain I-activated, 249.63 ± 58.03 pN	Activation: 24 h (B cells), 1–5 d (T cell); single-cell interaction, ~12.8-min dwell time (T cell–dendritic cell under flow); AFM, minutes per cell; confocal imaging, 5–20 min	AFM: topography, adhesion force and nanoindentation; laser scanning confocal microscopy: 3D synapse imaging and Ca^2+^ flux; finite-element simulation: shear stress profiles in microchannels; custom image-analysis software (Fiji, Imaris, and R)	Lymph-node-mimetic microchannel (e.g., 100 μm in height) with fibronectin-coated dendritic cell monolayer; parallel laminar streams for different T cell populations; precise shear stress control (0.01–100 dynˑcm^−2^); real-time analysis of antigen-specific adhesion and detachment	[113–115]
**Platelet**	Venous flow, 1–10 dynˑcm^−2^ˑ arterial flow, 10–50 dynˑcm^−2^; arterioles (peak), ~60 dynˑcm^−2^;	Severe arterial stenosis, >500 dynˑcm^−2^; mechanical heart valves, up to 6,000 dynˑcm^−2^.	Resting: ~3 kPa and spreading: 15 kPa; thrombin-activated: mean elastic modulus softens from ~12.4 to ~7.5 kPa; local modulus ranges: 1–50 kPa (AFM)	Collagen-activated or acute myocardial infarction patient platelets, 2.18 MPa.	AFM: ~30 ms per curve (35 Hz); softening dynamics: thrombin-induced ~7.1 min; cytochalasin-D-induced ~2.7 min; activation stimulation: collagen treatment for ~2 h.	Hertz model (conical tip) for elastic-modulus extraction from AFM curves; numerical models for scanning ion conductance microscopy analysis	Glass-bottom dishes/coverslips for static adhesion/spreading assays; flow chambers for adhesion/aggregation under shear; geometrical constriction devices to mimic vascular stenosis	[116–118]

Note that the above table concisely summarizes key mechanical parameters (shear stress waveforms, stiffness ranges, exposure durations, simulation tools, and standard microfluidic designs), recommended reporting standards, and available public resources for blood cell mechanobiology experiments. The MechanoBase repository (https://compbio-zhanglab.org/mechanobase/) provides open-access datasets on cellular mechanics and microfluidic designs.

## Conclusion

**Fig. 4.**
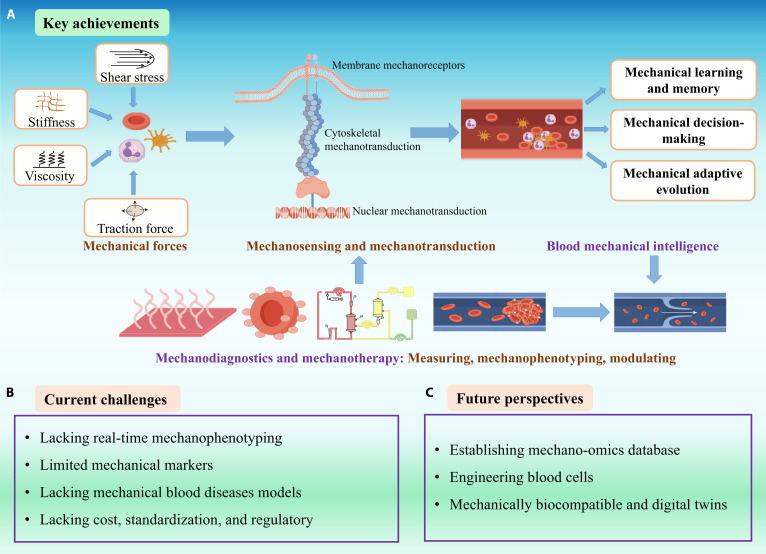
Achievements, challenges, and perspectives in blood mechanical intelligence. (A) Key achievements. Discovery of core mechanosensors (PIEZO1, integrins, and GPIb-IX-V), delineation of mechanical learning and memory, decision-making and adaptive evolution spanning membrane, cytoskeleton, nucleus, and first translational demonstrations in mechanodiagnostics and mechanotherapy. (B) Current challenges. Lack of real-time, in vivo force mechanophenotyping; incomplete biomarker thresholds that separate adaptation from pathology; limited disease-specific models; and lacking cost, standardization, and regulatory. (C) Future perspectives. We aim to develop mechano-omics atlases that pair mechanics with molecular programs, engineer cells for hostile flow, and deploy digital-twin guidance plus mechanically biocompatible devices to mitigate iatrogenic stress. Together, these steps operationalize blood’s mechanical intelligence for prevention, prognosis, and therapy.

Viewing blood as a force-interpreting system reframes hematology and vascular immunology. RBCs, leukocytes, and platelets do not merely endure hemodynamic load; they sense, integrate, store and act on mechanical information, with consequences for oxygen delivery, host defense, and hemostasis. Across this review, we synthesized evidence for mechanosensing, mechanical learning and memory, decision-making, and adaptive evolution and outlined how these properties can be measured and therapeutically modulated (Fig. 4A). However, some questions remain unresolved.

Challenges in clinical translation are as follows: (a) We still lack routine, in vivo, spatiotemporal readouts of forces and phenotypes at the cellular resolution; (b) the molecular inventory and biomarker thresholds that distinguish physiological from pathological mechanosense are incomplete, while simultaneously considering patient heterogeneity (age, sex, comorbidities, and device exposure history); (c) there are limited disease-specific models of “dysregulated blood mechanical intelligence” and a need for prospective validation; and (d) practical constraints include cost, standardization, and regulatory readiness for both devices and mechanotherapies (Fig. 4B). Multiple disciplines (physics, cell biology, engineering, and clinical medicine) are now converging to decode and eventually “hack” the body’s mechanical communication networks for better health outcomes. Below, we outline several key future directions that balance visionary ideas with emerging evidence in this field (Fig. 4C).

### Future perspectives

#### Distinguishing physiological from pathological mechanosensing

A near-term priority is to phenotype force–response set points and hysteresis for each blood cell lineage across health and disease. Some mechanical “memory” is adaptive (e.g., wear-mediated splenic culling of old RBCs), whereas persistent stiffening in metabolic disease reduces deformability and shortens lifespan, contributing to hemolysis [103]. Similarly, hypertension and aging synergize to amplify thrombus contractility and stiffness, suggesting that of clots (e.g., aggregate contractile force and elastic modulus) could stratify risk and guide targeted inhibition of GPVI and GPIbα or downstream tension sensors such as ROCK. Thus, establishing age-, sex-, and comorbidity-stratified reference ranges for RBC relaxation time, leukocyte elastic modulus, and platelet aggregate force would provide the field with actionable cutoffs for clinical trials.

#### “Mechano-omics”: Integrating mechanics with molecular state

A second opportunity is to formalize mechano-omics, integrating calibrated mechanical features with single-cell molecular profiles. Matrix stiffness reprograms hematopoietic stem/progenitor fate via YAP/TAZ and lineage regulators, and these transcriptional states are reversible when cells are returned to compliant niches [86,104]. Electroporation-based lipid bilayer assay for cell surface tension and transcriptomics now couples high-throughput single-cell mechanical readouts with transcriptomes, enabling discovery of gene programs that set membrane tension, cortical prestress, and adhesive thresholds [19]. Building multimodal atlases that fuse mechanics, transcriptomics and epigenetics across hematopoiesis could reveal druggable control points in mechanotransduction and generate companion diagnostics that match patients to mechanotherapies.

#### Engineering and preconditioning cells for the mechanical milieu

Specific sensors and effectors can encode mechanical intelligence, and they can also be tuned or trained. CRISPR-based modulation of PIEZO channels or integrins and ex vivo mechanical preconditioning (defined shear/stiffness exposure) may harden therapeutic cells against hostile hemodynamics or improve tissue homing. Proof of concept comes from rhesus factor D stealth RBCs, which preserve deformability yet evade alloimmunity in vivo [97], and from microfluidic training regimens that emulate arterial shear or interstitial poroelasticity for platelets and immune cells [85,87,105]. A next step is to codify dose–response curricula (waveform, amplitude, and duty cycle) that produce durable, safe gains in performance.

#### Mechanically biocompatible environments and digital twins

At device scales, the goal is to design the mechanical environment in vitro to maintain their optimal function. Pulsatile waveforms in ECMO and pumps improve microvascular perfusion and blunt inflammation relative to nonpulsatile flow [99]; endothelium-mimetic, antithrombotic coatings that suppress FXII contact activation reduce thrombosis without heavy anticoagulation [100,101]; and computational-fluid-dynamics/machine-learning-optimized flow paths in VADs minimize turbulence and stagnation, lowering predicted hemolysis and platelet activation [102]. Coupling these interventions to patient-specific digital twins, models that integrate RBC/platelet mechanics with vessel geometry and flow, can identify mechanical risk signatures and prospectively evaluate therapies [26,88,106].

In conclusion, decoding blood’s mechanical language will enable a new class of diagnostics and therapies that act on forces, sensors, and thresholds rather than solely biochemical ligands. As mechano-omics maps expand and mechanotherapies mature, this field is poised for breakthroughs—from developing mechanosensor and mechanotransduction targets to designing cell mechanical modification and blood-compatible devices. This will enable precision medicine based on mechanical information: achieving early disease detection, matching patients with precise mechanical signal targets, and modifying the hemodynamic environment to keep blood cells within safe force ranges. Collectively, these steps translate the biomechanical intelligence of blood into practical solutions for prevention, prognosis, and therapy.

### Outstanding questions

• Resetting memory: Which mechanical memories are reversible, on what timescales, and by which interventions (Ca^2+^ buffering, histone deacetylase inhibitors, and cytoskeletal drugs)?
• Causal stacks: Can we phenotype from sensor engagement, state variables, and policy with causal interventions (optogenetic adhesion and mechanopharmacology)?
• Generalization versus overfitting: Do cells overspecialize to pathological shear/stiffness and lose adaptability (e.g., device-exposed platelets)?
• Clinical end points: Which mechanical end points (e.g., thrombus contractile force and RBC deformability variance) are prognostic and actionable?
• Mechano-omics: How do mechanical phenotypes align with transcriptomic/epigenomic states in vivo (paired single-cell assays in patients)?
• Device ecology: What shear spectra are tolerable in ECMO/VAD without priming maladaptive memories?

## Methods

To systematically gather evidence for this review focusing on hematological cells (including RBCs, leukocytes, and platelets), mechanical intelligence and its related mechanisms, diagnosis, and therapy, a comprehensive literature search was performed in 3 electronic academic databases: PubMed, Web of Science and EMBASE. Key search terms were designed to cover the core themes of this review, including combinations of “mechanical sensing”, “mechanotransduction”, “cell intelligence”, “cell learning”, “cell memory”, “hematological cells”, “red blood cell/RBC”, “white blood cell (WBC)/leukocyte”, “platelet”, “hematological cells mechanical intelligence”, “mechanical learning”, “mechanical memory”, “mechanical decision”, “mechanical adaptive evolution”, “PIEZO1”, “mechanosensor”, “shear stress”, “cytoskeleton”, and “mechanical diagnosis”. The literature retrieval covered a time range from 1978 to 2026, ensuring the inclusion of foundational studies on hematocyte mechanical properties and the latest advances in mechanical intelligence research. Additional papers were identified by backward and forward citation chaining of the key sources listed in the reference list. After initial screening based on titles and abstracts to exclude irrelevant studies, full-text articles were reviewed to verify the validity and relevance of the evidence. Only peer-reviewed original research articles and authoritative review papers were included to ensure the reliability of the synthesized evidence. These studies comprehensively cover the mechanical intelligence characteristics of different hematocytes, their molecular regulatory mechanisms, and translational applications in mechanical diagnosis and therapy, providing a solid evidence base for the review.

## Data Availability

This study did not generate any new datasets. All data analyzed are from publicly available sources, as cited in the manuscript.
